# Supplementary material for: Are carbon emissions trading and green financial instruments synergistic? -Comprehensive quantitative research based on content analysis
Source: PLoS One. 2024 Mar 7;19(3):e0298601. doi: 10.1371/journal.pone.0298601 (PMC10919601; doi:10.1371/journal.pone.0298601)
Supplement: S1 Data — (ZIP) [file pone.0298601.s001.zip › Data/地级市Z_F工作报告.zip!/╝¬┴╓/░╫│╟╩╨/└┤╫╘╣1⁄2╓┌║┼í╛┬φ┐╦╩2╛▌═°í┐_2014.docx]

各位代表：

　　现在，我代表市政府，向大会作政府工作报告，请予审议，并请各位政协委员和与会同志提出意见。

一、2013年工作回顾

　　一年来，我们在省委、省政府和市委的正确领导下，在市人大、市政协的监督支持下，紧紧依靠广大人民群众，齐心协力，扎实奋进，较好地完成了市五届人大一次会议审议确定的各项任务，全市经济社会发展迈出坚实步伐。预计2013年，地区生产总值完成700亿元，增长13%。固定资产投资完成565亿元，增长21%。规模以上工业增加值完成180亿元，增长14.5%。全口径财政收入完成55亿元，增长13.2%；地方级财政收入完成36亿元，增长16.1%。社会消费品零售总额完成250.8亿元，增长13.3%。城镇居民人均可支配收入22500元，农民人均纯收入7000元，分别增长11.6%和13%。主要经济指标增速均高于全省平均水平，地区生产总值、规模以上工业增加值、全口径财政收入、固定资产投资4项指标增速位居全省前列。

　　——经济发展取得新成效。全市新开工3000万元以上项目308个，增长26.2%，其中亿元以上项目130个，10亿元以上项目12个。新增规模以上工业企业20户，新增产值超亿元工业企业9户，规模以上工业总产值完成560亿元，增长20.5%。粮食播种面积1207万亩，实施高光效技术示范面积31.2万亩、膜下滴灌面积80万亩，粮食生产喜获大丰收。畜禽饲养量达到4482万头（只），产值实现112.1亿元，增长16.6%。服务业增加值完成245亿元，增长13%。旅游收入完成30.6亿元，增长25%。

　　——基础建设实现新突破。白城机场项目可研得到批复，配套工程启动并部分竣工。长白铁路、白阿铁路和平齐线电气化改造项目完成前期工作。嫩丹高速镇赉至白城段全面开工。完成国道15.1公里和省道28.4公里干线维修、农村公路394.1公里建设任务。镇赉、通榆2座公铁立交桥完成主体工程。通榆至梨树500千伏输电线路开工建设。分洪入向、中小河流治理完成渠道整治94.2公里。加快市区、县城开发改造。“富民强村固基”工程示范村建设启动项目1066个。

　　——生态环境得到新改善。在全省率先实施河湖连通工程，连通水库泡塘32个，增加蓄水量5.6亿立方米，恢复湿地60万亩。启动“三年再造百万林、建设西部大屏障”活动，造林41万亩。新增草原工程围栏10万亩，人工种草、草场改良30万亩。实施土地整理复垦工程，新增耕地38万亩，复垦面积2138亩。推进“清水工程”、“蓝天工程”和“环保专项行动”，城市污水收集率、处理率分别达到67%和62%，空气质量优良天数占94.3%，达到国家二级标准要求。通榆向海被评为全国20个魅力湿地之一。大安嫩江湾被授予“国家湿地公园”称号。

　　——改革开放迈出新步伐。推进市县卫生局与计生委机构合并。洮北联社改制组建农村商业银行。开展“营改增”、土地收益保证贷款、农村土地承包经营权确权登记等试点。开发区对外开放、加快发展成效明显，白城工业园区被评为国家农业产业化示范基地，白城经济开发区跻身全省开发区十强。市领导驻点招商、“走出去”主题招商、利用“东博会”会展招商取得良好效果，与112家域外商会建立友好合作关系，同20多个国家和地区开展经贸交流。全市招商引资到位资金680亿元，增长26%。

　　——民生保障有了新提升。全市十个方面民生实事基本完成。城镇新增就业4.76万人，就业困难人员再就业4800人，城镇登记失业率3.74%。农村劳动力转移就业38.5万人（次）。城乡低保保障标准分别提高到每月336元、每年1800元。新农保、城居保共参保41.2万人。城镇职工和居民基本医疗保险、新农合实现全覆盖。改造棚户区234.2万平方米，建设廉租房27.3万平方米。拆除城市危房8433户。改造农村危房32468户。“暖房子”工程既有建筑节能改造197万平方米。维修改造校舍16.9万平方米，新建改建公办幼儿园6所，建设农村标准化学校6所。建设农村社区服务中心92个。新建的白城中心医院投入使用。城市医疗垃圾实现无害化处理。全民健身中心基本竣工。城乡文化基础设施建设实现“六个全覆盖”。解决6万农村人口饮水安全问题。8.5万农村贫困人口实现脱贫。

　　——社会事业得到新发展。全市组织申报省级以上科技计划项目88项。完成实用技术项目对接36项。县（市、区）全部通过国家科技进步考核。推广通榆义务教育均衡发展经验。全市7030名进城务工人员子女、2435名留守儿童平等接受义务教育。“歌舞鹤乡•放飞梦想”群众文化活动被列入国家公共文化服务体系示范项目。镇赉公立医院改革试点取得阶段性成果。“平安医院”创建活动深入开展，标准化村卫生室建设加快推进，重点传染病得到有效防控。计划生育优生“两免”项目全面实现。白城市居家养老示范中心项目开工建设。“军地对接、军民互助”活动成效显著。村委会第九次换届圆满完成。少数民族“百村万户”致富工程全面启动。外事侨务、广播电视、妇女儿童、残疾人、老龄、气象、人防、档案、地方志和红十字会等事业取得新进步。

　　——和谐稳定呈现新局面。“六五”普法扎实推进，法律服务“十百千”活动有效开展，纠纷调解成功率达到98%以上。积极探索网上信访有效途径，领导干部接访下访活动深入开展，信访积案化解率达到93%。行政复议工作进一步加强。城市报警监控系统不断完善，城乡治安防控网络建设强力推进，治安案件发案起数下降11.5%。保持无重大餐饮食品安全事故、无重大药品安全事件“双无”目标。完善地震预防体系，有效应对松原地震。公共突发事件得到及时有效处置。工矿商贸、道路交通和火灾“三类事故”发生起数下降29.7%，死亡人数下降20%。

　　——政府效能实现新提高。实行经济发展、城市建设、社会管理3个联席会议制度，加强会商研究和综合施策，推进了工作落实。创办科学行政讲堂，学习法规、研究政策、交流经验，提高了工作能力。首次对县（市、区）、开发区和市直部门实行分类绩效考核。认真办理人大代表建议38件、政协委员提案101件，办复率和满意率均达到100%。全面落实市人大、市政协视察调研提出的意见和建议。减少行政审批项目101项，压缩32%。机构编制总量实现“零增长”。阳光政务“六项制度”进一步完善。开展“三公”经费公开试点。坚决整治“四风一顽症”，机关作风建设年活动取得较好成效。全面落实“一岗双责”，廉政建设进一步加强。

　　一年来，我们围绕全局抓统筹，立足重点求突破，下大力气抓了以下七项工作：

　　（一）突出发展民营经济。研究制定了《加快民营经济发展的实施意见》，出台50条政策措施。建设中小企业创业孵化基地，组建中小企业服务中心，成立民营企业家协会。组织民营企业家赴浙江绍兴、山东潍坊等地学习考察。举办“名人讲坛”。开展民营经济“走进院校、走进社区、走进乡镇”活动。全市民营经济实现主营业务收入992亿元，增长21.3%；从业人员27万人，增长11.6%；上缴税金28亿元，增长11.6%。

　　（二）大力推进农业产业化。坚持“高起点、大集团、全链条”，引进新建通榆咱家集团5000万只肉鸡、镇赉维尔鸭业5000万只肉鸭、洮南雏鹰集团400万头生猪、大安龙兴公司130万头生猪、洮北亚亨公司100万只肉羊和白城工业园区中绿粗杂粮等一批产业化项目。扶持壮大白城经济开发区绿稼源等25户龙头企业，农产品加工业销售收入突破250亿元。新增农业合作社1588个、家庭农场248个。创建地方特色品牌，新增中国驰名商标1个、地理标志证明商标2个、吉林省著名商标18个、省级以上名牌产品9个。通榆“三千禾”品牌实现原产地网络直销。

　　（三）着力发展高载能产业。编制了《白城市风电本地消纳综合示范区实施方案》。搭建风电、火电、用电企业联动平台，探索建立“财政补贴+关联企业让利”运作模式，在白城金升镍业公司进行试点。引进洽谈富达棒材等高载能项目15个。白城云基地计算中心建成运营。启动建设大安两家子、白城工业园区高载能产业园。开展中丹可再生能源利用技术交流合作，探索清洁能源开发管理新模式。协调对接五大电力央企，实施清洁供暖试点。大安开发风电30万千瓦。洮南全晟50兆瓦太阳能项目实现并网发电。

　　（四）加快城市开发改造。生态新区新建续建道路10条，铺设管网58.8公里，鹤鸣湖工程完成土方85万立方米，棚户区改造回迁区开工面积20万平方米。老城区打造长庆街、丽江路2条标准化街路，新建、改造道路17条，改造东风道口、白城至侯家公路出口道路12.6公里。铺装人行步道和居民小区83万平方米。改造排水管网34公里、给水管网21公里，铺设燃气主干管网28.2公里。拆除市区违章建筑1020户、10万平方米。美化旧楼房103栋。规范广告牌匾3000多处。各县（市）加强了县城建设，打造了兴隆山、福顺、坦途、安广等一批特色小城镇。林海镇•生态新区被列入全省22个示范城镇。

　　（五）狠抓安全生产。按照省委、省政府和市委的要求部署，在全市开展了安全生产隐患大排查大整改活动，共成立47个督查组、1441个检查组，实行抓块全覆盖、抓条保重点、抓点盯关键，突出14个重点行业领域和42个重大隐患点，采取“六查、十检、四改”和“建档”有力措施，全市发现隐患16656处，整改16280处，停产整顿企业289户，关闭取缔企业87户。完成了煤矿企业停产整顿、危险化学品企业条件整顿、涉氨企业专项治理等重点任务。建立健全了以“网格化”为重点、以“标准化”为核心、以“信息化”为手段、以“常态化”为保障的安全生产长效监管机制。

　　（六）全力抗洪救灾。今年，我市遭遇了1998年以来最大洪水。嫩江、洮儿河300多公里堤防告急，水位超汛限运行长达91天。我们在省委、省政府和市委的正确领导下，实行市、县、乡、村四级联动，组织11.6万干部群众和部队官兵，开展了“防”、“抢”、“转”攻坚战，累计动用土石方190万立方米、投入机械设备1.28万台次，抢修堤防200多公里、抢护险工险段50多处，确保了月亮泡6号坝、嫩江回水堤、新荒泡4号坝、哈尔淖渔场、长山岱等重点险工险段安全，成功转移安置群众3195人。通过全市上下艰苦奋战，取得了人员无一伤亡、国堤无一决口、水库无一垮坝、群众财产损失降到最低的全面胜利。适时开展了抗灾保粮和灾后重建，新建住房480户、维修住房2248户，受灾群众生产生活得到妥善安置。

　　（七）实施十百工程。围绕市委的战略部署，注重抓载体、促落实。坚持工程化思维，谋划实施了百个扶贫项目攻坚、百个重大项目招商、百户重点企业培育、百项城市基础设施建设、百个收入增长点培育、百湖连通、百项实用技术对接、百个品牌推广、百名企业经营管理者培养、百名农村致富能人培养十百工程。坚持项目化分解，每项工程由百个项目（单元）作支撑，每个项目做到纵向到底、横向到边，滚动实施。坚持责任化落实，成立了工程领导小组，明确部门责任主体，任务到点、责任到人。坚持时序化推进，每个工程明确阶段性任务，实行年度考核。

　　各位代表！

　　这些成绩的取得来之不易，是省委、省政府和市委坚强领导、科学决策的结果，是市人大、市政协有效监督、全力支持的结果，是全市各族人民戮力同心、奋勇拼搏的结果。在这里，我代表白城市人民政府，向全市各族人民，各民主党派、工商联、人民团体、无党派人士、离退休老同志，向驻白的中省直部门和企事业单位、人民解放军指战员、武警部队官兵、公安司法干警，向关心和支持白城发展的各界朋友，致以崇高的敬意和衷心的感谢！

　　同时，我们也清醒地认识到，工作和发展中还存在许多问题和不足。主要是：经济发展具有拉动作用的大项目少，产业链条短、品牌附加值低；城市基础设施建设不完善，投融资渠道窄，市区管理体制不顺；信访维稳机制还不够健全，化解问题力度还不够大，长期积累的矛盾还没有从根本上得到解决；民生事业发展投入不足，公共服务设施薄弱，解决渠道单一。政府自身建设有待加强，少数部门和干部创新意识不强、作风不实、办事效率不高的现象还存在。对于这些问题，我们一定要高度重视，认真加以解决。

二、2014年工作安排

　　党的十八届三中全会作出了全面深化改革的重大战略部署，掀开了中国改革开放的新篇章，对全面建成小康社会将产生重大而深远的影响。围绕吉林全面深化改革，省委十届三次全会提出了16个方面改革意见和12个方面改革试点。市委五届七次全会立足白城实际，提出了研究谋划好改革、跟进落实好改革、创新实践好改革的新要求，为全面深化改革明确了主攻方向。

　　我们要抓住改革机遇，推动转型发展。利用国家实行生态建设最优惠的补偿政策和最严格的约束制度，充分挖掘我市独特生态资源优势，打好生态经济牌。一是推进治沙治碱、湿地草原修复和河湖连通等工程，加强生态保护；二是围绕绿色农业、清洁能源和生态旅游，大力开发生态产业；三是探索集约、绿色、低碳城市建设模式，倡导生态理念。以生态立概念、打品牌、塑形象，向生态要品质、要效益、要幸福。

　　我们要落实改革举措，释放市场活力。发挥市场在资源配置中的决定性作用，开展资源评估与规划，并注重与市场对接，实现资源价值最大化；放宽基础设施建设、公共服务等领域市场准入条件，引进社会资本，拓宽投融资渠道，实现投资主体多元化；落实改革措施，激活土地、金融、技术、人才等生产要素，并借助市场机制促进流动，实现要素配置最优化。

　　我们要把握改革趋势，转变政府职能。明确政府与市场的边界，把握简政放权趋势，把下放给地方的职能“接得好”，把放给市场和社会的权力“放到位”，把政府该管的事情“管得住”，严格行政审批管理，下移监管重心，创新治理方式，实现由全能政府向有限政府转变，加快建设法治政府和服务型政府。

　　我们要增强改革动力，激发奋进状态。破除落后保守思维，改变传统方式方法，强化自我革新、自我创新的思想观念，增强只争朝夕、时不我待的机遇意识，保持勇挑重担、坚韧不拔的工作干劲，凝聚群策群力、攻坚克难的智慧力量，营造昂扬向上、奋发有为的浓厚氛围，最大限度聚集改革正能量，打响全面深化改革攻坚战。

　　2014年是落实改革、创新发展的关键之年，政府工作总的要求是：深入贯彻党的十八大、十八届三中全会、中央经济工作会议精神，落实省委十届三次全会和市委五届七次全会的部署，围绕推动创新发展、统筹发展、绿色发展、开放发展、安全发展和建设活力、富裕、和谐、平安白城的要求，坚持以推进改革创新为统揽，以提升发展质量和民生福祉为目标，以转变政府职能和工作作风为保障，继续大力实施“三大战略”，全力打造“六大功能区”，努力开创白城改革发展的新局面。

　　全市经济社会发展的主要预期目标是：地区生产总值完成790亿元，增长10.6%。全口径财政收入完成62亿元，增长12.7%；地方级财政收入完成41亿元，增长13.9%。规模以上工业增加值完成207亿元，增长12%。固定资产投资完成678亿元，增长20%。社会消费品零售总额完成278亿元，增长10.8%。城镇居民人均可支配收入实现24900元、农民人均纯收入实现7750元，增长10.6%以上，与经济增长同步。城镇新增就业人数4.8万人以上，城镇登记失业率控制在4.5%以内，单位GDP能耗下降完成省定指标。

　　（一）千方百计加大投资。突出抓大项目。围绕“五业振兴”谋划大项目，利用富裕电力消纳政策引进大项目，依托开发区集聚大项目。全年组织建设超亿元项目200个以上，投资规模达到500亿元以上。抓好白城机场主体工程建设，争取嫩丹高速白城段全线、白阿铁路白城至镇西段、嫩江公路大桥开工。加强项目管理。围绕项目招得实、批得快、建得好，制定项目谋划、引进、审批、建设、管理办法，强化责任包保、跟踪服务、定期调度，促进项目早建设、早投产。创新招商策略。抢抓国家实施新一轮扩大开放契机，加强与发达地区、外埠开发区和商会的战略合作；推动我市企业与域外有资本、有技术、有品牌的优势企业嫁接合作；帮助企业分支机构争取总部在资金、市场、技术等方面给予更多倾斜。全年招商引资到位资金达到810亿元，增长20%。

　　（二）强力推进工业增长。鼓励多生成。探索民营经济在市场准入、市场监管、市场公平竞争、金融创新等方面的配套改革，发挥民营企业公共服务平台作用，加快6个中小企业创业孵化基地建设，培训创业者6000人（次），新增小微企业300户以上。扶持快成长。开展企业一对一帮扶行动，着力解决企业融资、用工、市场开拓等问题。实施百个技改项目，扶持30户企业进入规模以上行列。全年新增产值超亿元企业10户。推动早做强。通过嫁接重组、上市扩股、品牌培育、人才引进等措施，提高企业整体实力。培育产值超5亿元企业10户、超10亿元企业5户。加强增长管理。强化工业运行的监测调度，协调企业提高本地产品配套率，研究制定激励措施，推动企业增产增效。

　　（三）大力促进农业增效。推进规模化生产。制定土地适度规模经营的意见，土地流转力争突破100万亩，规模化生产面积达到190万亩。加快全程农机化进程。鼓励多元化经营。实施农民收入倍增计划，引导“一业为主、多种经营”，鼓励从事养殖、加工、商贸、流通、旅游等行业，多渠道增加农民财产性收入。突出精致化发展。发展节水农业、循环农业。围绕“六畜兴旺”、10个特色产业发展，建设20个农业示范园区，新改扩建200个标准化养殖场（小区）。发展棚膜经济，推动品种改良和反季生产。加强效益化管理。选育新品种，推广新技术，实施保护性耕作面积达到135万亩。加强田间管理，促进作物早熟。加强庭院管理，新增农户科学储粮仓1.15万套。抓好品牌化提升。实施“健康米、放心肉”工程，创建全国食源性食品安全生产基地。整合农畜产品特色品牌，利用各种平台，加大宣传营销力度。加强组织化建设。组织发展家庭农场、专业大户、农业合作社等新型农业经营主体，培育100个示范性农业合作社。

　　（四）积极推动服务业发展。做大商贸物流。加快编制商业网点布局、物流发展和电子商务规划。完善白城内陆港功能，开工建设建材家居物流产业园。新建改建农贸市场9个。创建国家级杂豆（绿豆）外贸出口示范基地。培育特色旅游。编制乡村旅游规划，打造“一线八点”精品旅游线路。加快向海、莫莫格湿地旅游开发，推进军事文化园开工、辽金文化城立项、城四家子城址考古和查干浩特旅游目的地建设。强化金融服务。启动“新三板”筹发工作。深入推进信用联社改制。发展村镇银行，组建农业专业担保公司，新增小额贷款公司10家。做大做实中兴公司、新开集团融资平台。积极探索林地、草场、水面等领域物权融资。研究开展粮食收储托管融资服务。加强信用体系建设。发展房地产业。加快解决历史遗留无籍房登记问题，规范房地产购销行为和房产中介服务，活跃二手房市场。

　　（五）加快推进城镇化。突出科学规划。编制白城特色城镇化总体规划，以法定形式进行刚性管理。注重提升质量。继续实施老城区改造，铺设地下管网103公里，实现2万燃气用户安全用气。新建垃圾中转站5座和垃圾发电项目。加快生态新区14条道路、2座立交桥、鹤鸣湖、条带水系等基础设施建设。推进碧桂园城市综合开发等产业项目。坚持示范引领。加快镇赉南湖新城、大安滨江新区、洮南北部新城和白城经济开发区二龙村新型社区建设，推进通榆铁西区、洮北铁东区改造。建设大屯、林海、舍力、瞻榆、黑水等一批特色小城镇。深入推进“富民强村固基”工程示范村建设。加强制度建设。深化户籍改革，推进农业转移人口市民化。探索建立被征地农民社会保障、进城落户农民养老保险和农村宅基地制度。加强进城农民职业培训，保障随迁子女义务教育。强化城市管理。坚持规划上移、管理下移的原则，理顺市区城市管理体制。开展卫生、秩序、违建整治行动。加强物业管理。

　　（六）突出加强生态修复。推进河湖连通。开挖维修渠道80公里，新连通水库泡塘29个。搞好分洪入向、大安灌区配套、引嫩入白供水延伸、洮儿河治理和灌区建设等工程，完成月亮泡蓄滞洪区前期工作。加快荒漠化治理。编制白城荒漠化治理规划。加大林地清收力度。深入开展“三年再造百万林、建设西部大屏障”活动，实施“三北”五期工程，完成造林35万亩。实施草原综合治理30万亩。抓好百万亩芦苇湿地工程建设。推进向海生态移民。实施吉林西部土地整理二期工程。强化环境保护。启动实施大气污染防治行动计划，推进重点流域水污染防治项目建设，完成主要污染物总量减排任务。落实最严格的水资源管理制度。

　　（七）全力实施扶贫攻坚。抓致富项目。坚持精准、有效扶贫，编制产业扶贫规划。推进百个扶贫项目建设。全年完成8万贫困人口脱贫。抓政策扶持。抢抓国家在东北和蒙东地区打造新的增长带契机，加大向上争取力度，整合国家和全省有关方面政策、项目、资金资源。抓设施建设。围绕“保基本”，推进水、电、路、房等基础设施建设，完成44个重点村整村推进任务。抓合力攻坚。实行省负总责、市级推进、县抓落实的工作机制。坚持政府扶持、社会帮扶与自力更生有效结合的工作原则。把提高扶贫对象生活水平作为主要考核指标。

　　（八）努力抓好民生改善。重点办好十个方面民生实事。一是扶持就业创业。培育打造创业促就业基地20个，城镇新增就业4.8万人，城镇登记失业率控制在4.5%以内，就业困难人员再就业4000人。二是抓好社会保险扩面。推进养老保险制度全覆盖，稳妥解决国企改制并轨人员失业保险问题，城镇居民基本医疗保险续保率达到100%。三是强化住房保障。改造棚户区254万平方米，建设廉租住房9万平方米，城市危房改造回迁安置房全部建成使用，改造农村危房3万户，“暖房子”既有建筑节能改造150万平方米。四是保障城乡饮水安全。改造扩建二水厂，关闭市区生活类自备水源井。新建农村水源工程23处，解决2万人口饮水安全问题。五是实施公交便民工程。新增、更新城市公共交通车辆70辆以上，新建候车亭30个。落实市区70周岁以上老年人免费乘坐公交车政策。六是推进居家养老服务。建成居家式养老服务中心示范小区300户。七是实施城乡教育普惠工程。新建改建公办幼儿园5所，建设农村标准化学校5所，维修改造校舍1.8万平方米。八是加强特困群体救助。建立部门联动覆盖城乡的社会救助体系。提高城乡居民最低生活保障标准和补助标准。为残疾人提供各类康复服务2万人（次）。完成农村妇女“两癌”筛查。九是完善城乡文体设施。建设社区文化活动室35个、农村健身路径70个。十是改善农村人居环境。实施以生活垃圾治理、饮用水源地保护、畜禽粪便处理为主要内容的农村环境综合整治工程。

　　（九）统筹发展社会事业。建立产学研协调机制。完成30项实用技术需求对接，抓好35个特色农产品品质鉴定和产业分析。推进义务教育均衡发展，整合职业教育资源，鼓励社会力量办学。加强民族教育。抓好十项文化惠民工程提升，推进省级文化产业试验园区、吉剧创作演出基地建设，打造“歌舞鹤乡•放飞梦想”白城群众文体活动品牌。深化基层医疗卫生机构综合改革。开展创建“三好一满意”医院活动。启动建设白城中医院、急救中心、儿童专科医院、结核病防治研究所、残疾人康复中心项目。探索政府向社会力量购买服务的方式方法。进一步落实计划生育家庭奖励扶助政策。搞好第三次全国经济普查。推动军民融合发展，深化国防教育改革，加强国防动员和后备力量建设，抓好双拥共建。开展精神文明创建活动。关爱空巢老人、特困残疾人和农村留守儿童。切实保护妇女和未成年人合法权益。

　　（十）切实维护社会稳定。强化安全监管防控体系建设，大力实施专项整治，坚决遏制重特大事故发生。完善信访工作制度，加大矛盾纠纷化解力度。创新行政复议方法。深入开展“法律六进”活动。推进“平安白城”建设，依法防范和惩治违法犯罪活动。推广社区网格化管理模式，加快社区公共事务服务中心建设。开展食品药品安全专项整治行动，加强食品药品检验检测能力建设。完善突发公共事件预警信息发布系统。健全防灾减灾救灾机制，加强救灾物资储备库建设。加大依法管理网络力度，提高舆论引导和应对能力。

　　（十一）加强政府自身建设。开展好党的群众路线教育实践活动，切实解决好“四风一顽症”问题。自觉接受人大法律监督、工作监督和政协民主监督，认真办理人大代表建议、意见和政协委员提案。深入实施十百工程，切实把发展、建设、民生任务落到实处。继续推行“三会一堂”制度，提高科学行政能力。加强政府性债务和优惠政策管理。强化审计监督。做好行政审批改革“接、放、管”工作。深化政务信息公开，拓展群众监督和舆论监督方式。全面履行“一岗双责”，认真落实党风廉政建设责任制和反腐倡廉建设新要求。强化行政执法、效能监督，切实加强政行风和软环境建设。严格执行《党政机关厉行节约反对浪费条例》等一系列规章制度。从严控制机构编制和行政公用经费。

　　各位代表！

　　改革催人奋进，发展任重道远。我们坚信：有省委、省政府和市委的正确领导，有市人大、市政协的大力支持，有全市上下的同心协力，一定能攻坚克难、乘势而上，圆满完成2014年的各项任务，继续朝着加快白城富民强市、全面建成小康社会的目标迈进！
